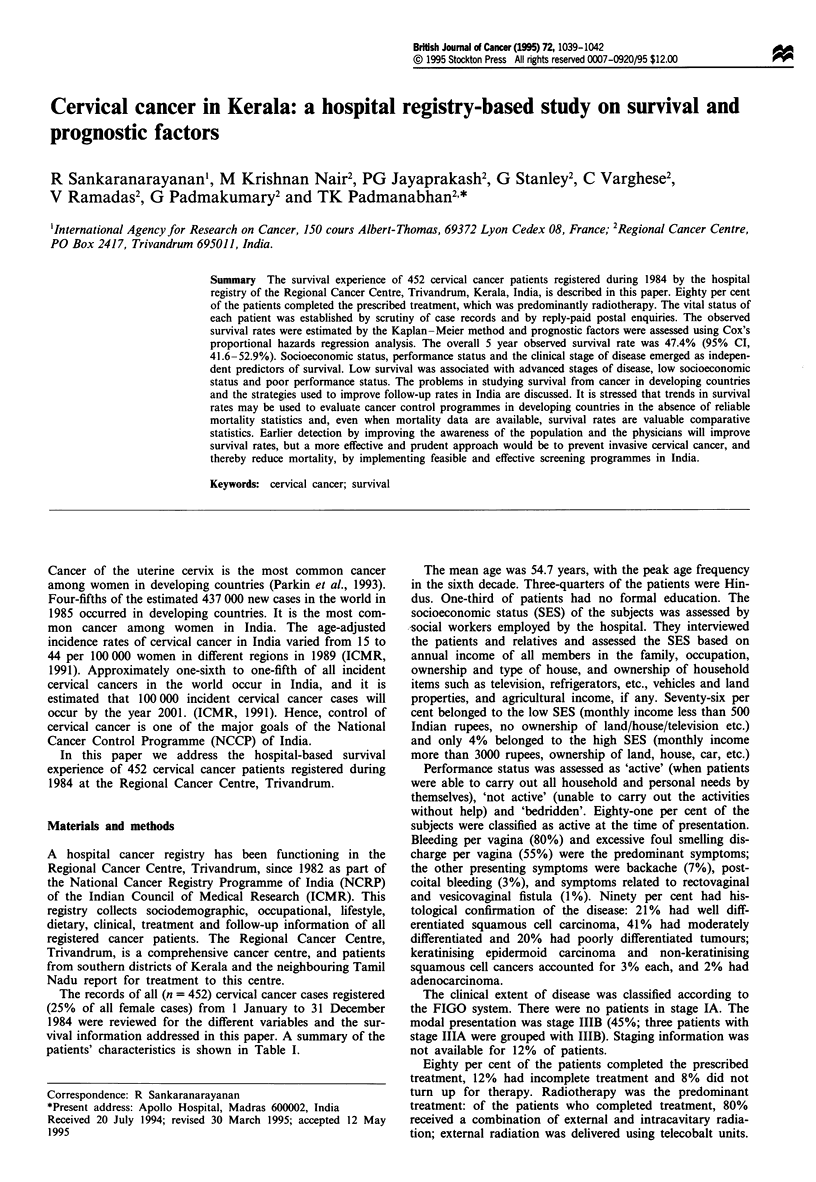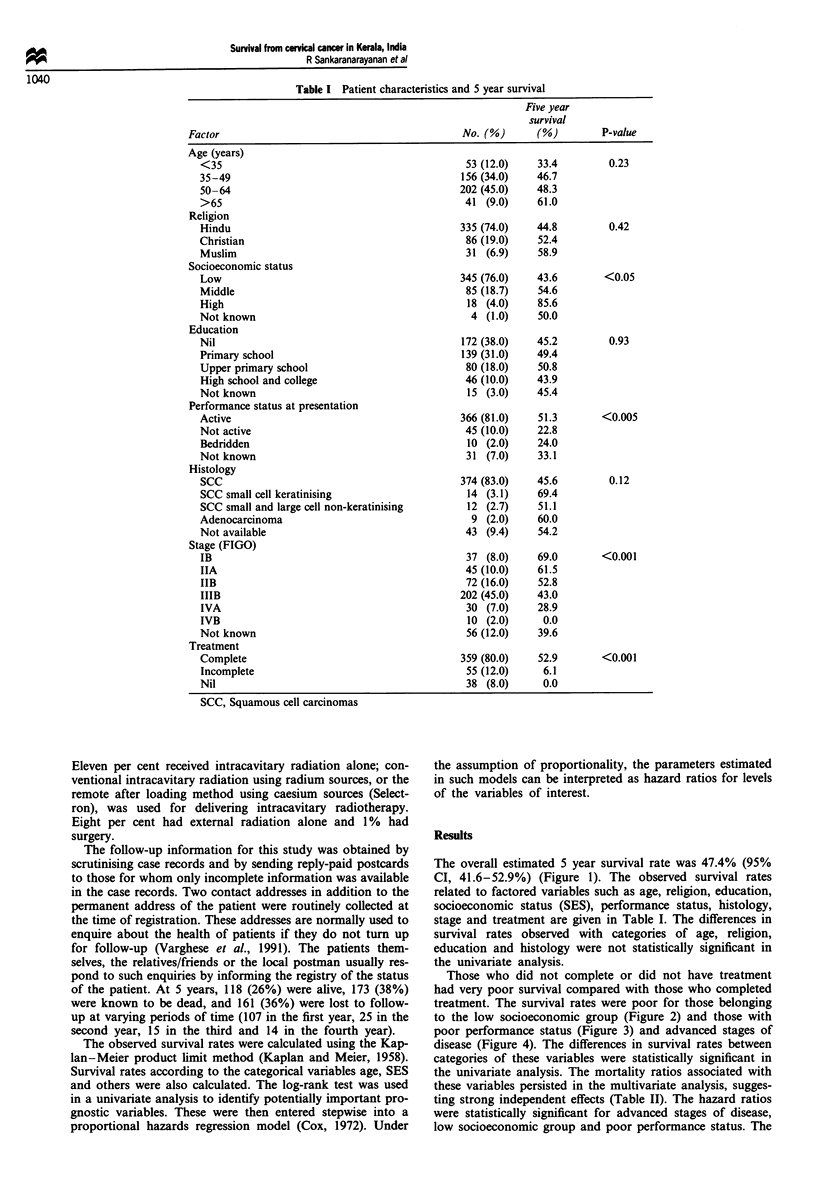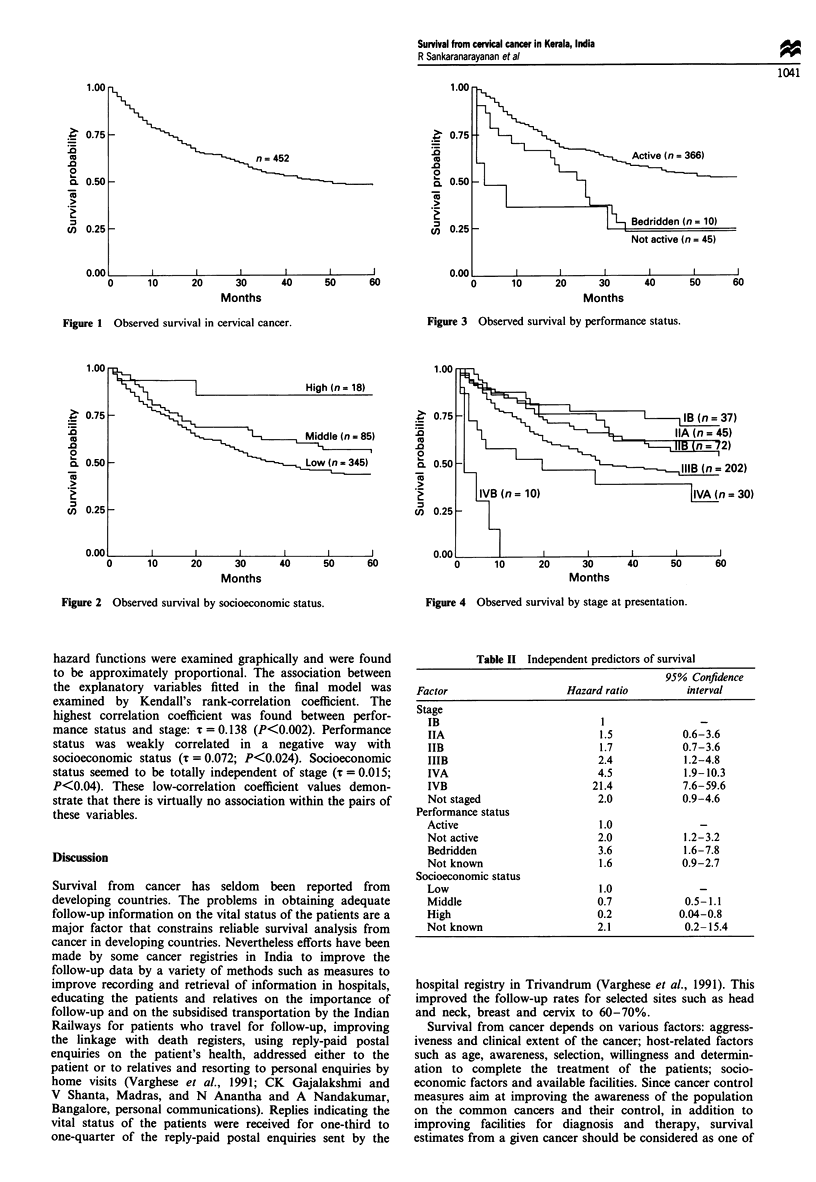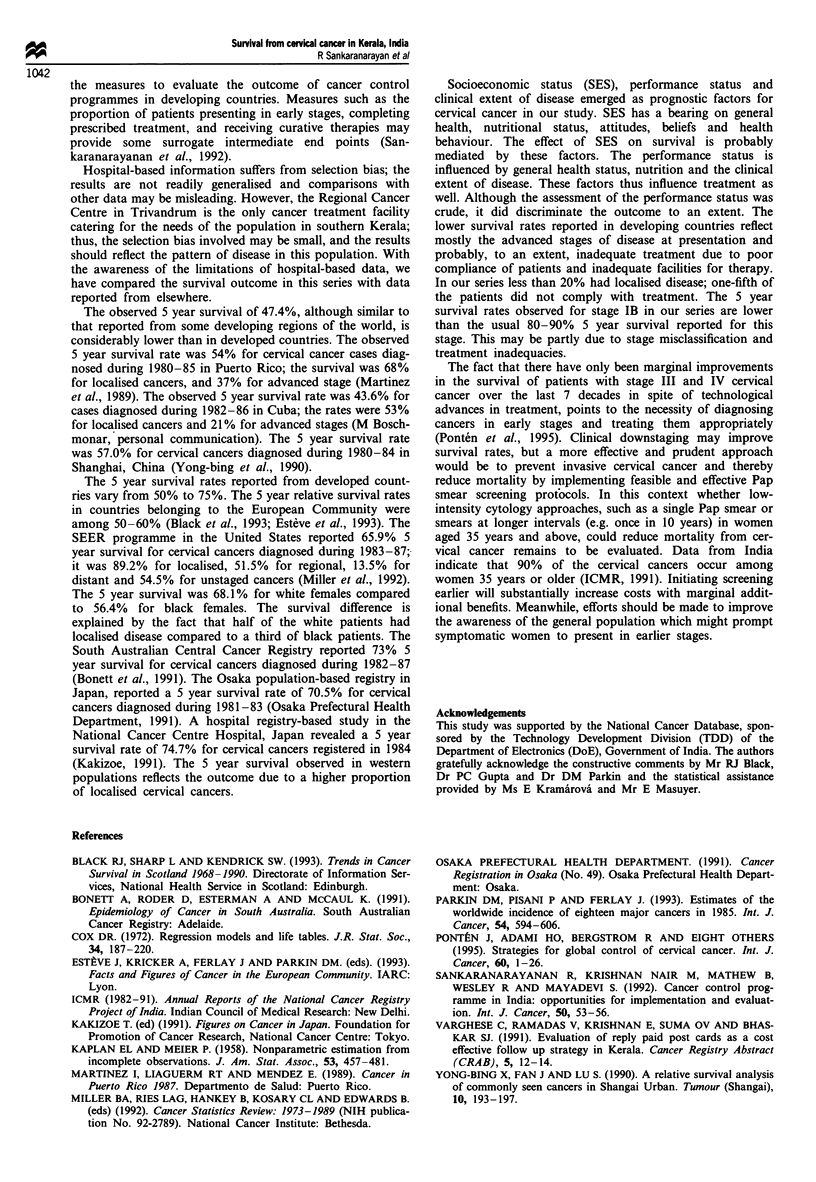# Cervical cancer in Kerala: a hospital registry-based study on survival and prognostic factors.

**DOI:** 10.1038/bjc.1995.458

**Published:** 1995-10

**Authors:** R. Sankaranarayanan, M. K. Nair, P. G. Jayaprakash, G. Stanley, C. Varghese, V. Ramadas, G. Padmakumary, T. K. Padmanabhan

**Affiliations:** International Agency for Research on Cancer, Lyon, France.

## Abstract

The survival experience of 452 cervical cancer patients registered during 1984 by the hospital registry of the Regional Cancer Centre, Trivandrum, Kerala, India, is described in this paper. Eighty per cent of the patients completed the prescribed treatment, which was predominantly radiotherapy. The vital status of each patient was established by scrutiny of case records and by reply-paid postal enquiries. The observed survival rates were estimated by the Kaplan-Meier method and prognostic factors were assessed using Cox's proportional hazards regression analysis. The overall 5 year observed survival rate was 47.4% (95% CI, 41.6-52.9%). Socioeconomic status, performance status and the clinical stage of disease emerged as independent predictors of survival. Low survival was associated with advanced stages of disease, low socioeconomic status and poor performance status. The problems in studying survival from cancer in developing countries and the strategies used to improve follow-up rates in India are discussed. It is stressed that trends in survival rates may be used to evaluate cancer control programmes in developing countries in the absence of reliable mortality statistics and, even when mortality data are available, survival rates are valuable comparative statistics. Earlier detection by improving the awareness of the population and the physicians will improve survival rates, but a more effective and prudent approach would be to prevent invasive cervical cancer, and thereby reduce mortality, by implementing feasible and effective screening programmes in India.


British Journal of Cancer (1995) 72, 1039–1042
© 1995 Stockton Press All rights reserved 0007-0920/95 $12.00

### Cervical cancer in Kerala: a hospital registry-based study on survival and prognostic factors

R Sankaranarayanan[1], M Krishnan Nair[2], PG Jayaprakash[2], G Stanley[2], C Varghese[2], V Ramadas[2], G Padmakumary[2] and TK Padmanabhan[2,*]

[1]*International Agency for Research on Cancer, 150 cours Albert-Thomas, 69372 Lyon Cedex 08, France;* [2]*Regional Cancer Centre, PO Box 2417, Trivandrum 695011, India.*

**Summary** The survival experience of 452 cervical cancer patients registered during 1984 by the hospital registry of the Regional Cancer Centre, Trivandrum, Kerala, India, is described in this paper. Eighty per cent of the patients completed the prescribed treatment, which was predominantly radiotherapy. The vital status of each patient was established by scrutiny of case records and by reply-paid postal enquiries. The observed survival rates were estimated by the Kaplan–Meier method and prognostic factors were assessed using Cox's proportional hazards regression analysis. The overall 5 year observed survival rate was 47.4% (95% CI, 41.6–52.9%). Socioeconomic status, performance status and the clinical stage of disease emerged as independent predictors of survival. Low survival was associated with advanced stages of disease, low socioeconomic status and poor performance status. The problems in studying survival from cancer in developing countries and the strategies used to improve follow-up rates in India are discussed. It is stressed that trends in survival rates may be used to evaluate cancer control programmes in developing countries in the absence of reliable mortality statistics and, even when mortality data are available, survival rates are valuable comparative statistics. Earlier detection by improving the awareness of the population and the physicians will improve survival rates, but a more effective and prudent approach would be to prevent invasive cervical cancer, and thereby reduce mortality, by implementing feasible and effective screening programmes in India.

**Keywords:** cervical cancer; survival

Cancer of the uterine cervix is the most common cancer among women in developing countries (Parkin *et al.*, 1993). Four-fifths of the estimated 437 000 new cases in the world in 1985 occurred in developing countries. It is the most common cancer among women in India. The age-adjusted incidence rates of cervical cancer in India varied from 15 to 44 per 100 000 women in different regions in 1989 (ICMR, 1991). Approximately one-sixth to one-fifth of all incident cervical cancers in the world occur in India, and it is estimated that 100 000 incident cervical cancer cases will occur by the year 2001. (ICMR, 1991). Hence, control of cervical cancer is one of the major goals of the National Cancer Control Programme (NCCP) of India.

In this paper we address the hospital-based survival experience of 452 cervical cancer patients registered during 1984 at the Regional Cancer Centre, Trivandrum.

### Materials and methods

A hospital cancer registry has been functioning in the Regional Cancer Centre, Trivandrum, since 1982 as part of the National Cancer Registry Programme of India (NCRP) of the Indian Council of Medical Research (ICMR). This registry collects sociodemographic, occupational, lifestyle, dietary, clinical, treatment and follow-up information of all registered cancer patients. The Regional Cancer Centre, Trivandrum, is a comprehensive cancer centre, and patients from southern districts of Kerala and the neighbouring Tamil Nadu report for treatment to this centre.

The records of all ($n = 452$) cervical cancer cases registered (25% of all female cases) from 1 January to 31 December 1984 were reviewed for the different variables and the survival information addressed in this paper. A summary of the patients' characteristics is shown in Table I.

Correspondence: R Sankaranarayanan
*Present address: Apollo Hospital, Madras 600002, India
Received 20 July 1994; revised 30 March 1995; accepted 12 May 1995

The mean age was 54.7 years, with the peak age frequency in the sixth decade. Three-quarters of the patients were Hindus. One-third of patients had no formal education. The socioeconomic status (SES) of the subjects was assessed by social workers employed by the hospital. They interviewed the patients and relatives and assessed the SES based on annual income of all members in the family, occupation, ownership and type of house, and ownership of household items such as television, refrigerators, etc., vehicles and land properties, and agricultural income, if any. Seventy-six per cent belonged to the low SES (monthly income less than 500 Indian rupees, no ownership of land/house/television etc.) and only 4% belonged to the high SES (monthly income more than 3000 rupees, ownership of land, house, car, etc.)

Performance status was assessed as 'active' (when patients were able to carry out all household and personal needs by themselves), 'not active' (unable to carry out the activities without help) and 'bedridden'. Eighty-one per cent of the subjects were classified as active at the time of presentation. Bleeding per vagina (80%) and excessive foul smelling discharge per vagina (55%) were the predominant symptoms; the other presenting symptoms were backache (7%), post-coital bleeding (3%), and symptoms related to rectovaginal and vesicovaginal fistula (1%). Ninety per cent had histological confirmation of the disease: 21% had well differentiated squamous cell carcinoma, 41% had moderately differentiated and 20% had poorly differentiated tumours; keratinising epidermoid carcinoma and non-keratinising squamous cell cancers accounted for 3% each, and 2% had adenocarcinoma.

The clinical extent of disease was classified according to the FIGO system. There were no patients in stage IA. The modal presentation was stage IIIB (45%; three patients with stage IIIA were grouped with IIIB). Staging information was not available for 12% of patients.

Eighty per cent of the patients completed the prescribed treatment, 12% had incomplete treatment and 8% did not turn up for therapy. Radiotherapy was the predominant treatment: of the patients who completed treatment, 80% received a combination of external and intracavitary radiation; external radiation was delivered using telecobalt units.

**Table I** Patient characteristics and 5 year survival

| *Factor* | *No. (%)* | *Five year survival (%)* | P-*value* |
|---|---|---|---|
| Age (years) | | | |
| <35 | 53 (12.0) | 33.4 | 0.23 |
| 35–49 | 156 (34.0) | 46.7 | |
| 50–64 | 202 (45.0) | 48.3 | |
| >65 | 41 (9.0) | 61.0 | |
| Religion | | | |
| Hindu | 335 (74.0) | 44.8 | 0.42 |
| Christian | 86 (19.0) | 52.4 | |
| Muslim | 31 (6.9) | 58.9 | |
| Socioeconomic status | | | |
| Low | 345 (76.0) | 43.6 | <0.05 |
| Middle | 85 (18.7) | 54.6 | |
| High | 18 (4.0) | 85.6 | |
| Not known | 4 (1.0) | 50.0 | |
| Education | | | |
| Nil | 172 (38.0) | 45.2 | 0.93 |
| Primary school | 139 (31.0) | 49.4 | |
| Upper primary school | 80 (18.0) | 50.8 | |
| High school and college | 46 (10.0) | 43.9 | |
| Not known | 15 (3.0) | 45.4 | |
| Performance status at presentation | | | |
| Active | 366 (81.0) | 51.3 | <0.005 |
| Not active | 45 (10.0) | 22.8 | |
| Bedridden | 10 (2.0) | 24.0 | |
| Not known | 31 (7.0) | 33.1 | |
| Histology | | | |
| SCC | 374 (83.0) | 45.6 | 0.12 |
| SCC small cell keratinising | 14 (3.1) | 69.4 | |
| SCC small and large cell non-keratinising | 12 (2.7) | 51.1 | |
| Adenocarcinoma | 9 (2.0) | 60.0 | |
| Not available | 43 (9.4) | 54.2 | |
| Stage (FIGO) | | | |
| IB | 37 (8.0) | 69.0 | <0.001 |
| IIA | 45 (10.0) | 61.5 | |
| IIB | 72 (16.0) | 52.8 | |
| IIIB | 202 (45.0) | 43.0 | |
| IVA | 30 (7.0) | 28.9 | |
| IVB | 10 (2.0) | 0.0 | |
| Not known | 56 (12.0) | 39.6 | |
| Treatment | | | |
| Complete | 359 (80.0) | 52.9 | <0.001 |
| Incomplete | 55 (12.0) | 6.1 | |
| Nil | 38 (8.0) | 0.0 | |

SCC, Squamous cell carcinomas

Eleven per cent received intracavitary radiation alone; conventional intracavitary radiation using radium sources, or the remote after loading method using caesium sources (Selectron), was used for delivering intracavitary radiotherapy. Eight per cent had external radiation alone and 1% had surgery.

The follow-up information for this study was obtained by scrutinising case records and by sending reply-paid postcards to those for whom only incomplete information was available in the case records. Two contact addresses in addition to the permanent address of the patient were routinely collected at the time of registration. These addresses are normally used to enquire about the health of patients if they do not turn up for follow-up (Varghese *et al.*, 1991). The patients themselves, the relatives/friends or the local postman usually respond to such enquiries by informing the registry of the status of the patient. At 5 years, 118 (26%) were alive, 173 (38%) were known to be dead, and 161 (36%) were lost to follow-up at varying periods of time (107 in the first year, 25 in the second year, 15 in the third and 14 in the fourth year).

The observed survival rates were calculated using the Kaplan–Meier product limit method (Kaplan and Meier, 1958). Survival rates according to the categorical variables age, SES and others were also calculated. The log-rank test was used in a univariate analysis to identify potentially important prognostic variables. These were then entered stepwise into a proportional hazards regression model (Cox, 1972). Under the assumption of proportionality, the parameters estimated in such models can be interpreted as hazard ratios for levels of the variables of interest.

### Results

The overall estimated 5 year survival rate was 47.4% (95% CI, 41.6–52.9%) (Figure 1). The observed survival rates related to factored variables such as age, religion, education, socioeconomic status (SES), performance status, histology, stage and treatment are given in Table I. The differences in survival rates observed with categories of age, religion, education and histology were not statistically significant in the univariate analysis.

Those who did not complete or did not have treatment had very poor survival compared with those who completed treatment. The survival rates were poor for those belonging to the low socioeconomic group (Figure 2) and those with poor performance status (Figure 3) and advanced stages of disease (Figure 4). The differences in survival rates between categories of these variables were statistically significant in the univariate analysis. The mortality ratios associated with these variables persisted in the multivariate analysis, suggesting strong independent effects (Table II). The hazard ratios were statistically significant for advanced stages of disease, low socioeconomic group and poor performance status. The

Figure 1 Observed survival in cervical cancer.

Figure 3 Observed survival by performance status.

Figure 2 Observed survival by socioeconomic status.

Figure 4 Observed survival by stage at presentation.

hazard functions were examined graphically and were found to be approximately proportional. The association between the explanatory variables fitted in the final model was examined by Kendall's rank-correlation coefficient. The highest correlation coefficient was found between performance status and stage: $\tau = 0.138$ ($P < 0.002$). Performance status was weakly correlated in a negative way with socioeconomic status ($\tau = 0.072$; $P < 0.024$). Socioeconomic status seemed to be totally independent of stage ($\tau = 0.015$; $P < 0.04$). These low-correlation coefficient values demonstrate that there is virtually no association within the pairs of these variables.

### Discussion

Survival from cancer has seldom been reported from developing countries. The problems in obtaining adequate follow-up information on the vital status of the patients are a major factor that constrains reliable survival analysis from cancer in developing countries. Nevertheless efforts have been made by some cancer registries in India to improve the follow-up data by a variety of methods such as measures to improve recording and retrieval of information in hospitals, educating the patients and relatives on the importance of follow-up and on the subsidised transportation by the Indian Railways for patients who travel for follow-up, improving the linkage with death registers, using reply-paid postal enquiries on the patient's health, addressed either to the patient or to relatives and resorting to personal enquiries by home visits (Varghese *et al.*, 1991; CK Gajalakshmi and V Shanta, Madras, and N Anantha and A Nandakumar, Bangalore, personal communications). Replies indicating the vital status of the patients were received for one-third to one-quarter of the reply-paid postal enquiries sent by the

**Table II** Independent predictors of survival

| *Factor* | *Hazard ratio* | *95% Confidence interval* |
|---|---|---|
| Stage | | |
| IB | 1 | – |
| IIA | 1.5 | 0.6–3.6 |
| IIB | 1.7 | 0.7–3.6 |
| IIIB | 2.4 | 1.2–4.8 |
| IVA | 4.5 | 1.9–10.3 |
| IVB | 21.4 | 7.6–59.6 |
| Not staged | 2.0 | 0.9–4.6 |
| Performance status | | |
| Active | 1.0 | – |
| Not active | 2.0 | 1.2–3.2 |
| Bedridden | 3.6 | 1.6–7.8 |
| Not known | 1.6 | 0.9–2.7 |
| Socioeconomic status | | |
| Low | 1.0 | – |
| Middle | 0.7 | 0.5–1.1 |
| High | 0.2 | 0.04–0.8 |
| Not known | 2.1 | 0.2–15.4 |

hospital registry in Trivandrum (Varghese *et al.*, 1991). This improved the follow-up rates for selected sites such as head and neck, breast and cervix to 60–70%.

Survival from cancer depends on various factors: aggressiveness and clinical extent of the cancer; host-related factors such as age, awareness, selection, willingness and determination to complete the treatment of the patients; socioeconomic factors and available facilities. Since cancer control measures aim at improving the awareness of the population on the common cancers and their control, in addition to improving facilities for diagnosis and therapy, survival estimates from a given cancer should be considered as one of the measures to evaluate the outcome of cancer control programmes in developing countries. Measures such as the proportion of patients presenting in early stages, completing prescribed treatment, and receiving curative therapies may provide some surrogate intermediate end points (Sankaranarayanan et al., 1992).

Hospital-based information suffers from selection bias; the results are not readily generalised and comparisons with other data may be misleading. However, the Regional Cancer Centre in Trivandrum is the only cancer treatment facility catering for the needs of the population in southern Kerala; thus, the selection bias involved may be small, and the results should reflect the pattern of disease in this population. With the awareness of the limitations of hospital-based data, we have compared the survival outcome in this series with data reported from elsewhere.

The observed 5 year survival of 47.4%, although similar to that reported from some developing regions of the world, is considerably lower than in developed countries. The observed 5 year survival rate was 54% for cervical cancer cases diagnosed during 1980–85 in Puerto Rico; the survival was 68% for localised cancers, and 37% for advanced stage (Martinez et al., 1989). The observed 5 year survival rate was 43.6% for cases diagnosed during 1982–86 in Cuba; the rates were 53% for localised cancers and 21% for advanced stages (M Boschmonar, personal communication). The 5 year survival rate was 57.0% for cervical cancers diagnosed during 1980–84 in Shanghai, China (Yong-bing et al., 1990).

The 5 year survival rates reported from developed countries vary from 50% to 75%. The 5 year relative survival rates in countries belonging to the European Community were among 50–60% (Black et al., 1993; Estève et al., 1993). The SEER programme in the United States reported 65.9% 5 year survival for cervical cancers diagnosed during 1983–87; it was 89.2% for localised, 51.5% for regional, 13.5% for distant and 54.5% for unstaged cancers (Miller et al., 1992). The 5 year survival was 68.1% for white females compared to 56.4% for black females. The survival difference is explained by the fact that half of the white patients had localised disease compared to a third of black patients. The South Australian Central Cancer Registry reported 73% 5 year survival for cervical cancers diagnosed during 1982–87 (Bonett et al., 1991). The Osaka population-based registry in Japan, reported a 5 year survival rate of 70.5% for cervical cancers diagnosed during 1981–83 (Osaka Prefectural Health Department, 1991). A hospital registry-based study in the National Cancer Centre Hospital, Japan revealed a 5 year survival rate of 74.7% for cervical cancers registered in 1984 (Kakizoe, 1991). The 5 year survival observed in western populations reflects the outcome due to a higher proportion of localised cervical cancers.

Socioeconomic status (SES), performance status and clinical extent of disease emerged as prognostic factors for cervical cancer in our study. SES has a bearing on general health, nutritional status, attitudes, beliefs and health behaviour. The effect of SES on survival is probably mediated by these factors. The performance status is influenced by general health status, nutrition and the clinical extent of disease. These factors thus influence treatment as well. Although the assessment of the performance status was crude, it did discriminate the outcome to an extent. The lower survival rates reported in developing countries reflect mostly the advanced stages of disease at presentation and probably, to an extent, inadequate treatment due to poor compliance of patients and inadequate facilities for therapy. In our series less than 20% had localised disease; one-fifth of the patients did not comply with treatment. The 5 year survival rates observed for stage IB in our series are lower than the usual 80–90% 5 year survival reported for this stage. This may be partly due to stage misclassification and treatment inadequacies.

The fact that there have only been marginal improvements in the survival of patients with stage III and IV cervical cancer over the last 7 decades in spite of technological advances in treatment, points to the necessity of diagnosing cancers in early stages and treating them appropriately (Pontén et al., 1995). Clinical downstaging may improve survival rates, but a more effective and prudent approach would be to prevent invasive cervical cancer and thereby reduce mortality by implementing feasible and effective Pap smear screening protocols. In this context whether low-intensity cytology approaches, such as a single Pap smear or smears at longer intervals (e.g. once in 10 years) in women aged 35 years and above, could reduce mortality from cervical cancer remains to be evaluated. Data from India indicate that 90% of the cervical cancers occur among women 35 years or older (ICMR, 1991). Initiating screening earlier will substantially increase costs with marginal additional benefits. Meanwhile, efforts should be made to improve the awareness of the general population which might prompt symptomatic women to present in earlier stages.

**Acknowledgements**
This study was supported by the National Cancer Database, sponsored by the Technology Development Division (TDD) of the Department of Electronics (DoE), Government of India. The authors gratefully acknowledge the constructive comments by Mr RJ Black, Dr PC Gupta and Dr DM Parkin and the statistical assistance provided by Ms E Kramárová and Mr E Masuyer.

### References

BLACK RJ, SHARP L AND KENDRICK SW. (1993). *Trends in Cancer Survival in Scotland 1968–1990*. Directorate of Information Services, National Health Service in Scotland: Edinburgh.

BONETT A, RODER D, ESTERMAN A AND MCCAUL K. (1991). *Epidemiology of Cancer in South Australia*. South Australian Cancer Registry: Adelaide.

COX DR. (1972). Regression models and life tables. *J.R. Stat. Soc.*, **34**, 187–220.

ESTÈVE J, KRICKER A, FERLAY J AND PARKIN DM. (eds). (1993). *Facts and Figures of Cancer in the European Community*. IARC: Lyon.

ICMR (1982–91). *Annual Reports of the National Cancer Registry Project of India*. Indian Council of Medical Research: New Delhi.

KAKIZOE T. (ed) (1991). *Figures on Cancer in Japan*. Foundation for Promotion of Cancer Research, National Cancer Centre: Tokyo.

KAPLAN EL AND MEIER P. (1958). Nonparametric estimation from incomplete observations. *J. Am. Stat. Assoc.*, **53**, 457–481.

MARTINEZ I, LIAGUERM RT AND MENDEZ E. (1989). *Cancer in Puerto Rico 1987*. Departmento de Salud: Puerto Rico.

MILLER BA, RIES LAG, HANKEY B, KOSARY CL AND EDWARDS B. (eds) (1992). *Cancer Statistics Review: 1973–1989* (NIH publication No. 92-2789). National Cancer Institute: Bethesda.

OSAKA PREFECTURAL HEALTH DEPARTMENT. (1991). *Cancer Registration in Osaka* (No. 49). Osaka Prefectural Health Department: Osaka.

PARKIN DM, PISANI P AND FERLAY J. (1993). Estimates of the worldwide incidence of eighteen major cancers in 1985. *Int. J. Cancer*, **54**, 594–606.

PONTÉN J, ADAMI HO, BERGSTROM R AND EIGHT OTHERS (1995). Strategies for global control of cervical cancer. *Int. J. Cancer*, **60**, 1–26.

SANKARANARAYANAN R, KRISHNAN NAIR M, MATHEW B, WESLEY R AND MAYADEVI S. (1992). Cancer control programme in India: opportunities for implementation and evaluation. *Int. J. Cancer*, **50**, 53–56.

VARGHESE C, RAMADAS V, KRISHNAN E, SUMA OV AND BHASKAR SJ. (1991). Evaluation of reply paid post cards as a cost effective follow up strategy in Kerala. *Cancer Registry Abstract (CRAB)*, **5**, 12–14.

YONG-BING X, FAN J AND LU S. (1990). A relative survival analysis of commonly seen cancers in Shangai Urban. *Tumour* (Shangai), **10**, 193–197.